# The combination of breast cancer PDO and mini‐PDX platform for drug screening and individualized treatment

**DOI:** 10.1111/jcmm.18374

**Published:** 2024-05-09

**Authors:** Yuxin Cui, Ran Ran, Yanyan Da, Huiwen Zhang, Meng Jiang, Xin Qi, Wei Zhang, Ligang Niu, Yuhui Zhou, Can Zhou, Xiaojiang Tang, Ke Wang, Yu Yan, Yu Ren, Danfeng Dong, Yan Zhou, Hui Wang, Jin Gong, Fang Hu, Shidi Zhao, Huimin Zhang, Chengsheng Zhang, Jin Yang

**Affiliations:** ^1^ Precision Medicine Center, The First Affiliated Hospital of Xi'an Jiaotong University Xi'an Shaanxi People's Republic of China; ^2^ Cancer Center, The First Affiliated Hospital of Xi'an Jiaotong University Xi'an Shaanxi People's Republic of China; ^3^ Department of Medical Oncology The First Affiliated Hospital of Xi'an Jiaotong University Xi'an Shaanxi People's Republic of China; ^4^ Center for Molecular Diagnosis and Precision Medicine The First Affiliated Hospital, Jiangxi Medical College, Nanchang University Nanchang Jiangxi China; ^5^ Department of Breast Surgery The First Affiliated Hospital of Xi'an Jiaotong University Xi'an Shaanxi People's Republic of China

## Abstract

The majority of advanced breast cancers exhibit strong aggressiveness, heterogeneity, and drug resistance, and currently, the lack of effective treatment strategies is one of the main challenges that cancer research must face. Therefore, developing a feasible preclinical model to explore tailored treatments for refractory breast cancer is urgently needed. We established organoid biobanks from 17 patients with breast cancer and characterized them by immunohistochemistry (IHC) and next generation sequencing (NGS). In addition, we in the first combination of patient‐derived organoids (PDOs) with mini‐patient‐derived xenografts (Mini‐PDXs) for the rapid and precise screening of drug sensitivity. We confirmed that breast cancer organoids are a high‐fidelity three‐dimension (3D) model in vitro that recapitulates the original tumour's histological and genetic features. In addition, for a heavily pretreated patient with advanced drug‐resistant breast cancer, we combined PDO and Mini‐PDX models to identify potentially effective combinations of therapeutic agents for this patient who were alpelisib + fulvestrant. In the drug sensitivity experiment of organoids, we observed changes in the PI3K/AKT/mTOR signalling axis and oestrogen receptor (ER) protein expression levels, which further verified the reliability of the screening results. Our study demonstrates that the PDO combined with mini‐PDX model offers a rapid and precise drug screening platform that holds promise for personalized medicine, improving patient outcomes and addressing the urgent need for effective therapies in advanced breast cancer.

## 1 INTRODUCTION

According to the latest statistics released by the International Centre for Cancer in 2020, breast cancer surpassed lung cancer as the most commonly diagnosed cancer and the leading cause of cancer death among women worldwide.^1^ Breast cancer is a highly heterogeneous disease that consists of many different subtypes, which exhibit substantial differences in clinicopathological features, biological behaviour and gene expression profiles. Drug resistance, recurrence and metastasis are the main causes of treatment failure in most breast cancer patients. Over time, approximately 30% of patients initially diagnosed with earlier‐stage breast cancer will eventually develop recurrent or metastatic disease.^2^ These patients are prone to develop resistance to most of the standard treatment options, resulting in poor prognosis, with a 5‐year survival rate of only approximately 21%.^3^ The aggressive nature and high mortality rate of recurrent or metastatic breast cancer underline the need for more precise treatment of advanced breast cancer.

Cell lines and patient‐derived xenografts (PDXs) have been widely used in cancer research. However, PDOs have emerged as promising preclinical models for human cancers.^4^ Cancer cell lines are derived from a single clone, lacking the diverse cellular composition found in patient tumours.^5^ PDXs can simulate tumour growth conditions well and retain the biological behaviour and therapeutic response of the parental tumour, but the long culture cycle, low success rate and high cost make them unsuitable for guiding individualized clinical medication and high‐throughput drug sensitivity screening.^6^ Organoids are microscopic self‐organizing, 3D structures that can be generated from both normal and tumour tissues. PDO models offer advantages in capturing tumour heterogeneity, maintaining genetic stability, enabling personalized medicine approaches, facilitating preclinical drug development, and achieving a higher success rate of establishment.^7^ PDOs can be generated from a wide range of cancer types and subtypes, allowing for the creation of extensive PDO biobanks that can support various research studies. Mini‐PDX is a novel, rapid and accurate in vivo drug sensitivity test model that not only shortens the cycle of animal experiments in vivo to 1 week, but also effectively and accurately predicts patients' clinical responses to targeted therapy and chemotherapy.^8^ The mini‐PDX models were established by injecting patient‐derived tumour cells into hollow fibre capsules and implanting them under the skin of mice. Compared with traditional PDX models, is a time‐saving model with a higher success rate and strong clinical feasibility. So far, this model has been applied to a variety of solid tumours: gastric cancer,^8^, ^9^ ovarian cancer,^10^, ^11^ gallbladder cancer,^12^ pancreatic cancer,^13^ etc.

It is well known that relying solely on in vivo or in vitro drug sensitivity test data may not support the authenticity and reliability of results. Therefore, in this study, we not only established a breast cancer organoid biobank containing different molecular subtypes but also explored a combined PDO and mini‐PDX drug‐sensitive screening platform and applied it to a heavily pretreated advanced breast cancer patient to provide her with sensitive therapeutic options. Our case studies demonstrate that this integrated platform has the potential to guide rapid and effective personalized treatment for patients with refractory advanced breast cancer.

## 2 MATERIALS AND METHODS

### 2.1 Human specimens

Breast cancer tissues were obtained from patients undergoing surgical resection or biopsy at the First Affiliated Hospital of Xi'an Jiaotong University. The studies were conducted in accordance with recognized ethical guidelines. Samples were confirmed to be tumours based on pathological assessment. Informed consent was obtained prior to the acquisition of samples from all donors. The clinical information of patients was obtained from the medical records system and is summarized in Table S1 (*n* = 29). All surgically resected samples were separated into three parts for histology examination, genomic sequencing, and organoid generation. Similar to the surgically resected samples, biopsy tissues with lengths over 30 mm (1 mm diameter) were separated into three parts, while smaller samples were used for organoid generation only.

### 2.2 Human breast tumour organoid culture

For human tumour samples, tissues were minced and digested with 2–3 mg/mL collagenase (Sigma, C9407) in basal medium at 37°C for a maximum of 1–2 h. The cell suspensions were then filtered through a 70 μm cell strainer and centrifuged for 5 min at 1500 rpm. The pellet was washed in cold Advanced DMEM/F12 (Gibco, 12,634,010), and then was embedded in growth factor reduced Matrigel (Corning, 354,230), gels were solidified at 37°C for 15 min and cultured in human complete medium^14^ advanced DMEM/F12, HEPES 10 mM (Gibco, 15,630,080), Glutamax 1x (Gibco, 35,050,061), Penicillin/Streptomycin 1x (HyClone, SV30010), B27 1x (Gibco, 12,587,010), A83‐01500 nM (Tocris, 2939/10), human epidermal growth factor (hEGF) 50 ng/mL (Peprotech, AF‐100‐15‐1000), Noggin 100 ng/mL (Peprotech, 120‐10C‐100), human fibroblast growth factor (hFGF)‐7 5 ng/mL (PeproTech, 100–19‐10), hFGF‐10,100 ng/mL (PeproTech, 100–26‐25), N‐acetylcysteine 1.25 mM (Sigma‐Aldrich, A9165‐5G), Nicotinamide 10 mM (Sigma‐Aldrich, N0636‐100G), Y27632 10 μM (Abmole Bioscience, M1817), R‐spondin1 250 ng/mL (Sino Biological, 11,083‐HNAS), Neuregulin1 5 nM (Peprotech, 100–03‐100), SB202190 500 nM (Sigma‐Aldrich, S7067‐25MG). Culture media were replaced every 3–4 days. Passaging was performed every 1–3 weeks based on organoid density and size organoids were treated with TrypLE™ Express (Gibco, 12,605,010) for 15 min at 37°C. After digestion, an appropriate volume of basic medium was added to stop the digestion, centrifuged at 4°C and 1500 rpm for 5 min, washed with Advanced DMEM/F12, and spun down again. The cells were resuspended in Matrigel at a ratio of 1:3, seeded in the plates and cultured as described above. For storage, the organoids were dissociated and resuspended in recovery cell culture freezing medium (Gibco, 12,648,010) and frozen according to standard procedures.

### 2.3 Immunohistochemistry

Tissue was fixed in neutral buffered formalin and organoids were fixed in 4% paraformaldehyde, followed by dehydration, paraffin embedding and sectioning. A standard IHC protocol was followed to stain the tumour tissue samples using human monoclonal antibodies against ERα (Abcam, ab16660, 1:300), androgen receptor (AR) (CST, 5153 T, 1:100), PR (Abcam, ab101688, 1:300), HER2 (CST, 4290S, 1:400) and Ki‐67 (Proteintech, 27,309‐1‐AP, 1:100). Briefly, 5 μm paraffin‐embedded tissue sections or organoids were deparaffinized with xylene and dehydrated through different concentrations of alcohols, and endogenous peroxidase activity was quenched with 30% H_2_O_2_ in methanol for 30 min during dehydration. Then, slides were subjected to antigen retrieval using 10 mM sodium citrate repair fluid for 15 min in boiled water. After blocking for 15 min, slides were incubated with the respective human primary antibody at 4°C overnight, followed by incubation with a reaction amplifier for 10 min. After washing with 1 × phosphate buffered saline (PBS), slides were incubated with the respective conjugated anti‐rabbit or mouse secondary antibody for another 15 min. After washing, slides were incubated with 3, 3′‐diaminobenzidine tetrahydrochloride (DAB), when the colour changed and immediately washed with water. Next, slides were stained with haematoxylin for 3 min. Finally, sections were dehydrated and made transparent in increasing concentrations of ethanol and xylene, and covered with neutral resin.

### 2.4 Next generation sequencing

NGS was performed by ShiHe Gene Company and described as previously reported.^15^ Briefly, 1 μg of fragmented genomic DNA underwent end‐repairing, A‐tailing and ligation with indexed adapters sequentially, followed by size selection using Agencourt AMPure XP beads (Beckman Coulter). Hybridization‐based target enrichment was carried out with the GeneseeqOne™ pan‐cancer gene panel (425 cancer‐relevant genes, Geneseeq Technology Inc.), and xGen Lockdown Hybridization and Wash Reagents Kit (Integrated DNA Technologies). Libraries captured by Dynabeads M‐270 (Life Technologies) were amplified in KAPA HiFi HotStart ReadyMix (KAPA Biosystems) and quantified by qPCR using the KAPA Library Quantification kit (KAPA Biosystems). Target‐enriched libraries were sequenced on the HiSeq4000 platform (Illumina) with 2 × 150 bp paired‐end reads.

### 2.5 Cell viability was measured using CellTiter‐Glo

The cell viability of PDOs was measured using a CellTiter‐Glo (CTG) (Promega, G9681) cell proliferation kit. Briefly, PDOs were seeded in 10 μL of Matrigel in 96‐well plates (3000–6000 cells per well) for 3 days and then treated with culture medium containing different drugs (Table 1) at various concentrations. After 3 days, the supernatant was replaced with new medium containing drugs. After an additional 3 days of treatment, cell viability was detected using CTG according to the manufacturer's protocol. The luminescence was measured using a GloMax DISCOVER measurer and the data were analysed using GraphPad Prism 8.0.

**TABLE 1:** Drug list.

Drug	Supplier
Alpelisib	Selleck (S2814)
Taselisib	Selleck (S7103)
Buparlisib	Selleck (S2247)
Everolimus	Selleck (S1120)
Temsirolimus	Selleck (S1044)
Rapamycin	Selleck (S1039)
AZD8055	Selleck (S1555)
Palbociclib	Selleck (S1116)
Flavopiridol	Selleck (S1230)
Trilaciclib	Selleck (S8389)
Eprenetapopt	Selleck (S7724)
COTI‐2	Selleck (S8580)
Fulvestrant	Selleck (S1191)

### 2.6 Combination effect

In this experiment, CompuSyn software developed based on the Chou‐Talalay algorithm was used to calculate the joint index of alpelisib and fulvestrant. The calculation formula is CI=D1/DX1 + D2/DX2 + D1 × D2/DX1 × DX2. D1 and D2 are the respective concentrations required to produce the X effect when the two drugs are used in combination, DX1 and DX2 are the two drugs used alone to produce X The respective concentration required for the effect. When CI >1, the two compounds have an antagonistic effect; when CI = 1, the two compounds have an additive effect; and when CI <1, the two compounds have a synergistic effect.

### 2.7 Western blot

Organoids were incubated with alpelisib, fulvestrant or a combination for 6 days and then washed with cold 1 × PBS. These cells were suspended in RIPA lysis solution (Biyuntian, P0013C) containing 1% protein and phosphorylation inhibitor (Abmole bioscience, M5293) on ice for 30 min. Then, the cells were centrifuged at 13,000 g at 4°C for 15 min and the supernatants were collected. The concentration was detected by the bicinchoninic acid (BCA) concentration detection kit (Biyuntian, P0012). The remaining parts were mixed in 5× loading buffer (Biyuntian, P0015), boiled for 10 min in a metal water bath, and then subjected to sodium dodecyl sulfate‐polyacrylamide gel electrophoresis (SDS‐PAGE). After electrophoresis, proteins were transferred onto polyvinylidene fluoride (PVDF) membranes and incubated against primary antibody for 24 h. Membranes were washed with tris buffer saline‐Tween (TBS‐T) and incubated with a 1:5000 dilution of secondary antibody for 2 h. Protein bands were then visualized by Ultra Luminol/Enhancer Reagent (New Cell & Molecular, China). The following antibodies were used: PI3K‐110α (Abcam, ab1678, 1:500), p‐AKT (CST, 4060S, 1:1000), AKT (CST, 4691S, 1:1000), p‐mTOR (CST, 5536S, 1:1000), mTOR (CST, 2983S, 1:1000), p‐S6 (CST, 4857S, 1:1000), ER (CST, 13258S, 1:500) and GAPDH (Proteintech, 60,004‐1‐Ig, 1:10000).

### 2.8 Generation of mini‐patient‐derived xenograft (mini‐PDX)

Six‐ to eight‐week‐old female nude mice (BALB/cJGpt‐Foxn1^nu^/Gpt) were purchased from Jicuiyaokang Company. Mice were housed and maintained in laminar flow cabinets under a specific pathogen‐free environment in accordance with the current standards and regulations of the government. First, hollow fibre tubes (Repligen Shanghai Biological Company, S9320101) were cut into 7–8 cm pieces and the tubes were wetted in 100% ethyl alcohol for 30 min. The alcohol was removed and the tubes were sterilized. The hollow tubes were filled with the tumour cell suspensions using 1 mL syringe and every 1.5 cm seal with hot smooth‐jawed needle holders (20,000 cells/1.5 cm tube). The mice were anaesthetised using an isoflurane small animal anaesthesia machine (Rayward Life Technology Company). The prepared 1.5 cm hollow fibre tubes were implanted subcutaneously into the back of mice (one on each side). After 7 days of drug treatment, the cell viability of hollow fibres was detected by CTG assay.

### 2.9 Statistical analysis

All data and statistical analyses were performed by Statistical Package for the Social Sciences (SPSS) version 20.0 software (SPSS Inc) and GraphPad Prism 8.0 software (GraphPad Software Inc. USA). For multiple comparisons, one‐way ANOVA was conducted, which was normally distributed and homoscedastic; otherwise, the Kruskal‐Wallis test was used. In addition, Bonferroni correction was used. All data are presented as the mean ± SEM. A *p* value <0.05 was considered significant (*p < 0.05; **p < 0.01; ***p < 0.001).

## 3 RESULTS

### 3.1 Establishment of breast cancer organoid biobanks

**FIGURE 1:**
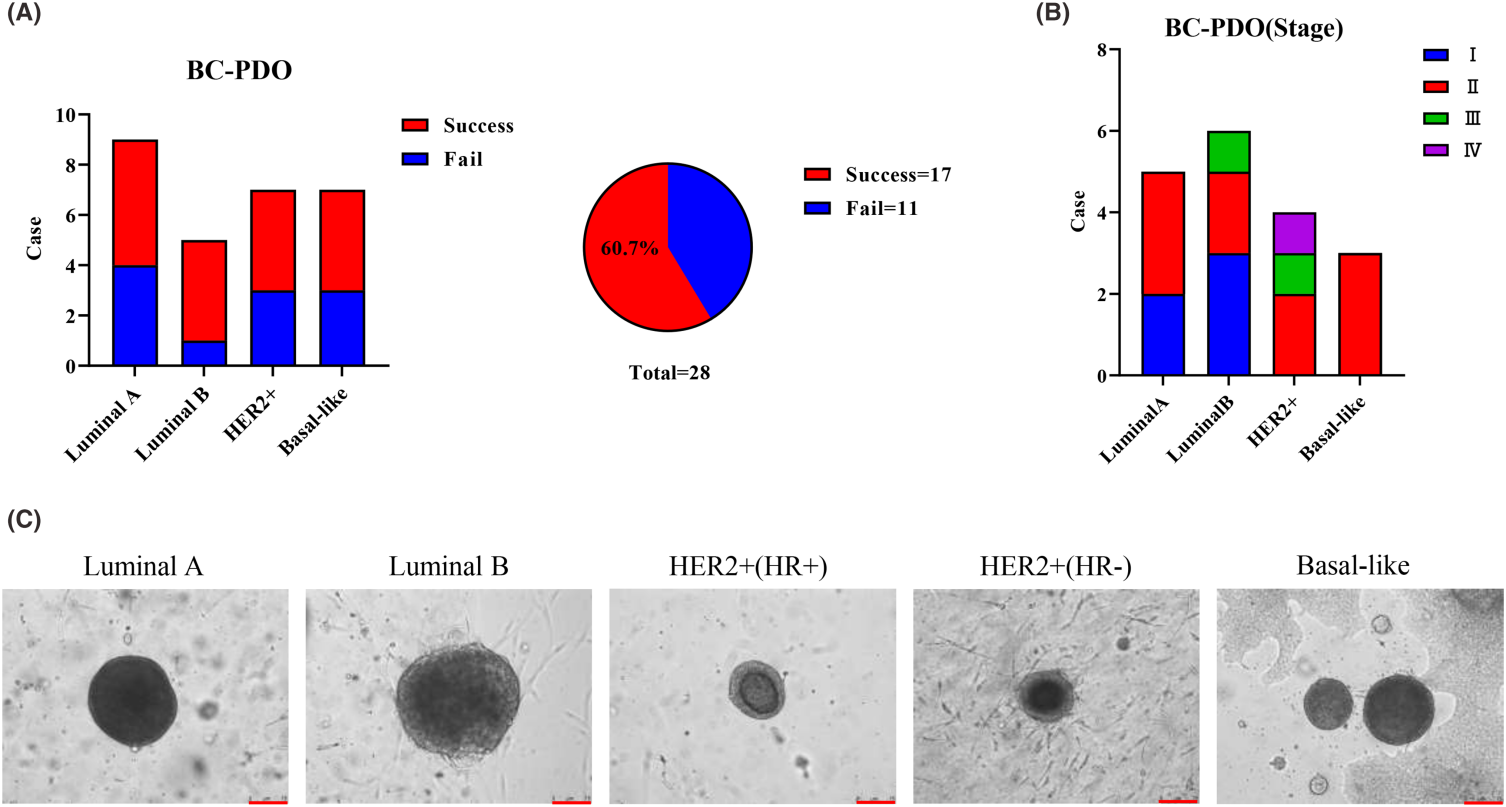
Establishment of breast cancer organoid biobanks for different molecular subtypes. (A) Statistical plot of the success rate of breast cancer organoids with different molecular subtypes. (B) Statistical plot of the staging of breast cancer organoids of different molecular subtypes. (C) Representative microscopic bright‐field images of breast cancer organoids with different molecular classifications, Scale bar = 75 μm.

To explore personalized drug therapy, we established a PDO biobank from patients' breast tumour tissue. A total of 29 samples were collected, including 28 cases of breast cancer and one case of benign fibroadenoma (Table S1). Seventeen cases of breast cancer organoids with different stages (Figure 1B) and molecular types (Figure 1A) were successfully established: For luminal A (5/9), luminal B (4/5), HER2+ (4/7) and basal‐like (4/7) the success rate of PDOs was approximately 60% (17/28) (Figure 1A), slightly lower than that of previous studies.^6^, ^14^, ^16^ This may be the result of our different definitions of successful culture. Our definition of successful culture is that the organoids can be continuously cultured for more than six generations because we observed that the probability of PDO failure increased significantly with increasing generations. Related studies have also shown that cell senescence exists at any growth stage of PDOs,^17^ which is the reason for the continuous optimization of culture conditions. Morphological observation showed that different molecular types of breast cancer PDOs all displayed cystic or solid structures and the central part of PDOs had a compact small cavity similar to the structure of breast ducts (Figure 1C), which was consistent with previous reports.^14^, ^16^, ^18^ In summary, we successfully established 17 breast cancer organoid biobanks containing different stages and molecular subtypes, representing the main subtypes of breast cancer which also provides a new tool for the future individualized precision treatment of breast cancer.

### 3.2 Breast cancer organoids retain the histological characteristics of parental tissues

**FIGURE 2:**
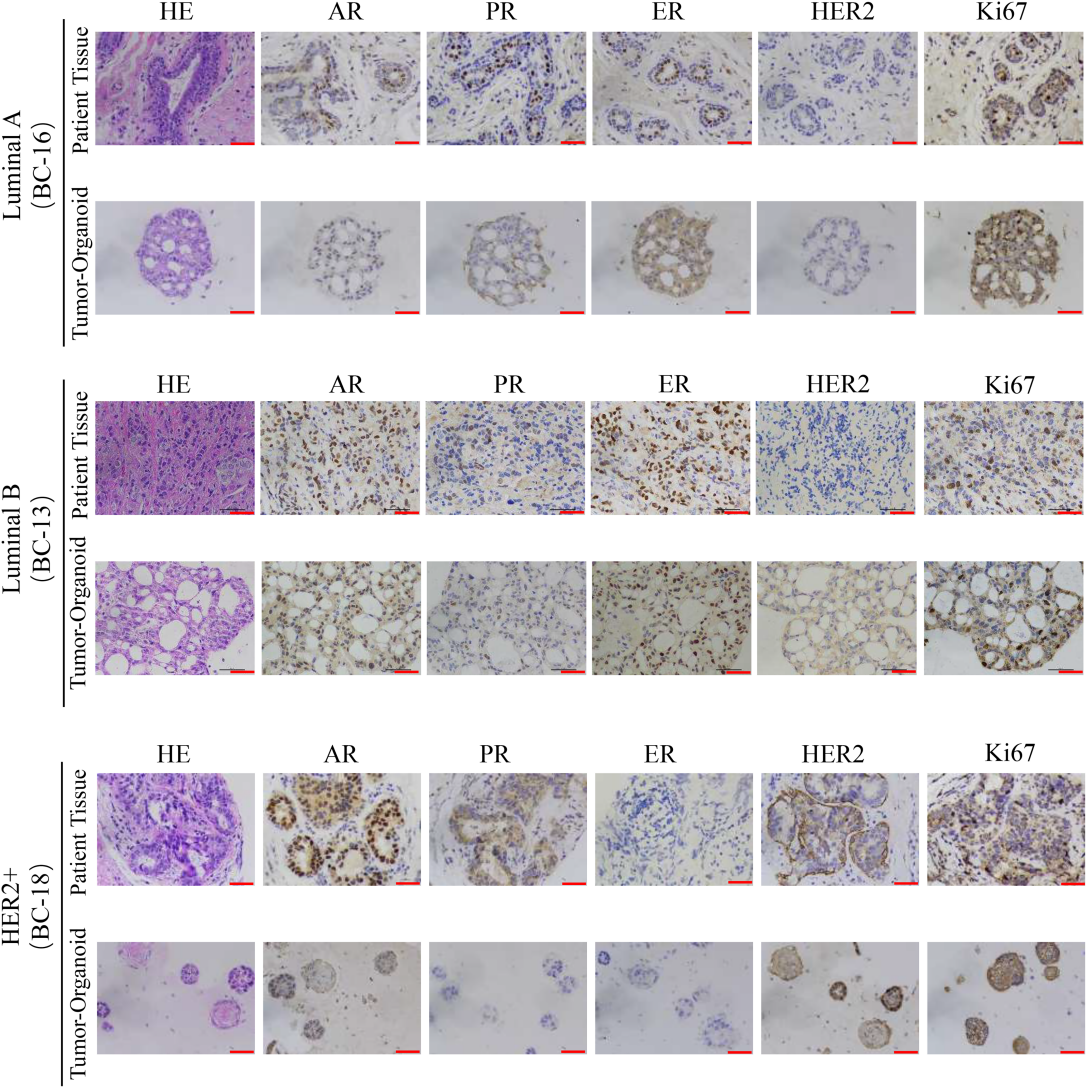
Histopathological characteristics of tumour tissues and organoids derived from breast cancer. HE and IHC staining of different types of breast tumour tissues and benign fibroadenoma compared with corresponding organoids. AR, PR, ER, HER2, Ki‐67; Scale bar = 50 μm.

We performed histopathological analysis of haematoxylin–eosin (HE) stained tissues and PDOs and confirmed that the phenotypes of organoids matched the original histological type of breast cancer. Based on the growth patterns and cellular and nuclear atypia, tumour‐derived organoids exhibit the same malignant characteristics as their parental tissues, including high density of cell populations, enlarged and irregular nuclei, and disappearance of the ratio of nuclear to plasma distribution (Figure 2). In addition to histological conservation, a typical breast cancer model should maintain expression of the most important and prevalent breast cancer biomarkers: ER, PR, HER2 and Ki‐67. The molecular subtype of breast cancer is mainly based on receptor status and the choice of treatment is largely dependent on the molecular subtype.^19^, ^20^ The status of hormone receptor (HR) has predictive value for endocrine therapy of breast cancer,^21^ while HER2 status not only can predict the outcome of systemic chemotherapy but is also a key factor in HER2‐targeted therapy.^22^ We found that the expression pattern of breast cancer biomarkers was well preserved in the breast cancer organoids as determined by IHC (Figure 2). However, we observed that a few PDOs did not inherit the HR or HER2 status of the original tissue, which may be related to the spatial heterogeneity of the tumour and the patients receiving neoadjuvant therapy. Overall, we found that the majority of breast cancer PDOs matched the original tumours in terms of histopathology as well as HR and HER2 status, and could be used as a high‐fidelity model for breast cancer.

### 3.3 Genetic characteristics of breast cancer organoids and parental tissues

**FIGURE 3:**
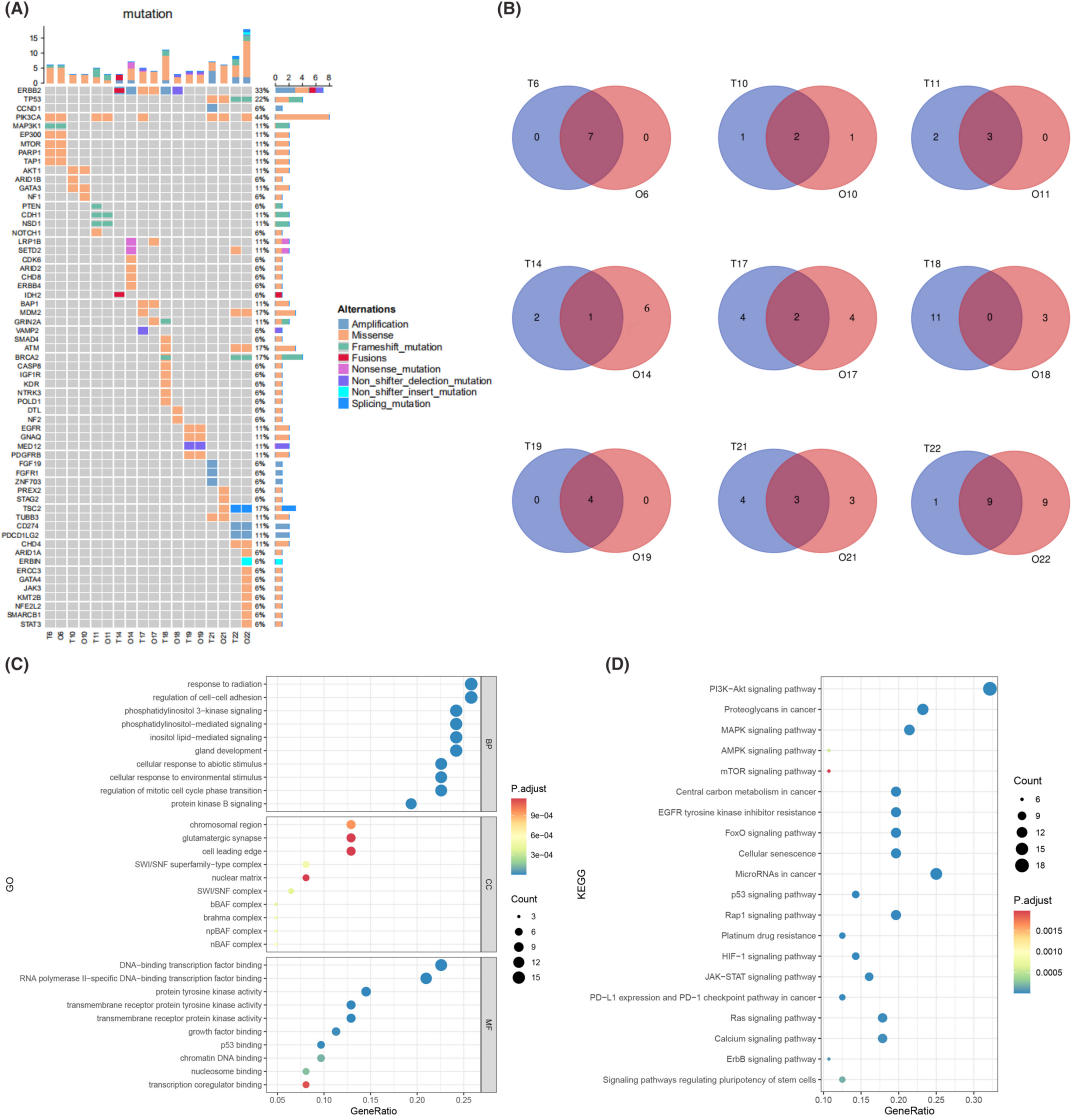
Breast cancer organoids recapitulate the genetic characterization of parental tumours. **(**A) Heatmap comparing the somatically mutated cancer genes in breast cancer organoids with those in parental tumour tissues. (B) The Venn diagram shows that the tumour tissues and organoids have many of the same mutations but also have different gene mutations. (C) GO enrichment pathway analysis diagram. (D) KEGG enrichment pathway analysis diagram. BP (biological process), CC (cellular component), MF (molecular function). The length of horizontal graph represents the gene ratio. The depth of the colour represents the adjusted *p*‐value. The area of circle in the graph means gene counts.

NGS was performed in nine representative pairs of breast cancer organoids and parental tumours to determine whether the breast cancer organoids could maintain the parental genomic features (Figure 3A). We found that most single nucleotide variation (SNV) genes in original tumours were preserved in organoids, especially the common mutated genes in breast cancer, such as PI3KCA, ERBB2, TP53, BRCA2 and MDM2, which were well retained in most breast cancer organoids. The Venn diagram showed that there was a large area of overlap between parental tumour tissues and PDOs in more than half of the cases, especially in cases 6 and 19, which reached 100% and cases 10 and 11 also had more than 60% overlap. However, there are also opposite situations, such as case 18 with a 0% overlap rate, which is a low probability event (1/9) after all (Figure 3B). In these nine cases, the number of SNV genes in the PDOs was always the same or more than that in the parental tissues (7/9), which was mainly due to the increase in tumour mutation burden as the PDOs continued to grow and passaging. Moreover, organoids are mainly derived from cancer‐initiating cells (CICs) and cancer stem cells (CSCs), which continue to expand and lead to the accumulation of SNV,^23^ while there is a certain proportion of normal cells in the parent tumour tissue. Using gene ontology (GO) and Kyoto Encyclopedia of Genes and Genomes (KEGG) databases for pathway enrichment analysis of sequencing results, we found that the high‐frequency signalling pathways such as PI3K‐AKT, mTOR, AMPK, MAPK and P53 were consistent with common gene mutations in breast cancer (Figure 3C,D), which further indicated that PDOs retained the heredity of parental tissue and provided a basis for subsequent drug screening. In summary, PDOs can largely retain the genomic characteristics of parental tissues, which provides a reliable guarantee for subsequent basic tumour research and personalized drug screening.

### 3.4 Case report

**FIGURE 4:**
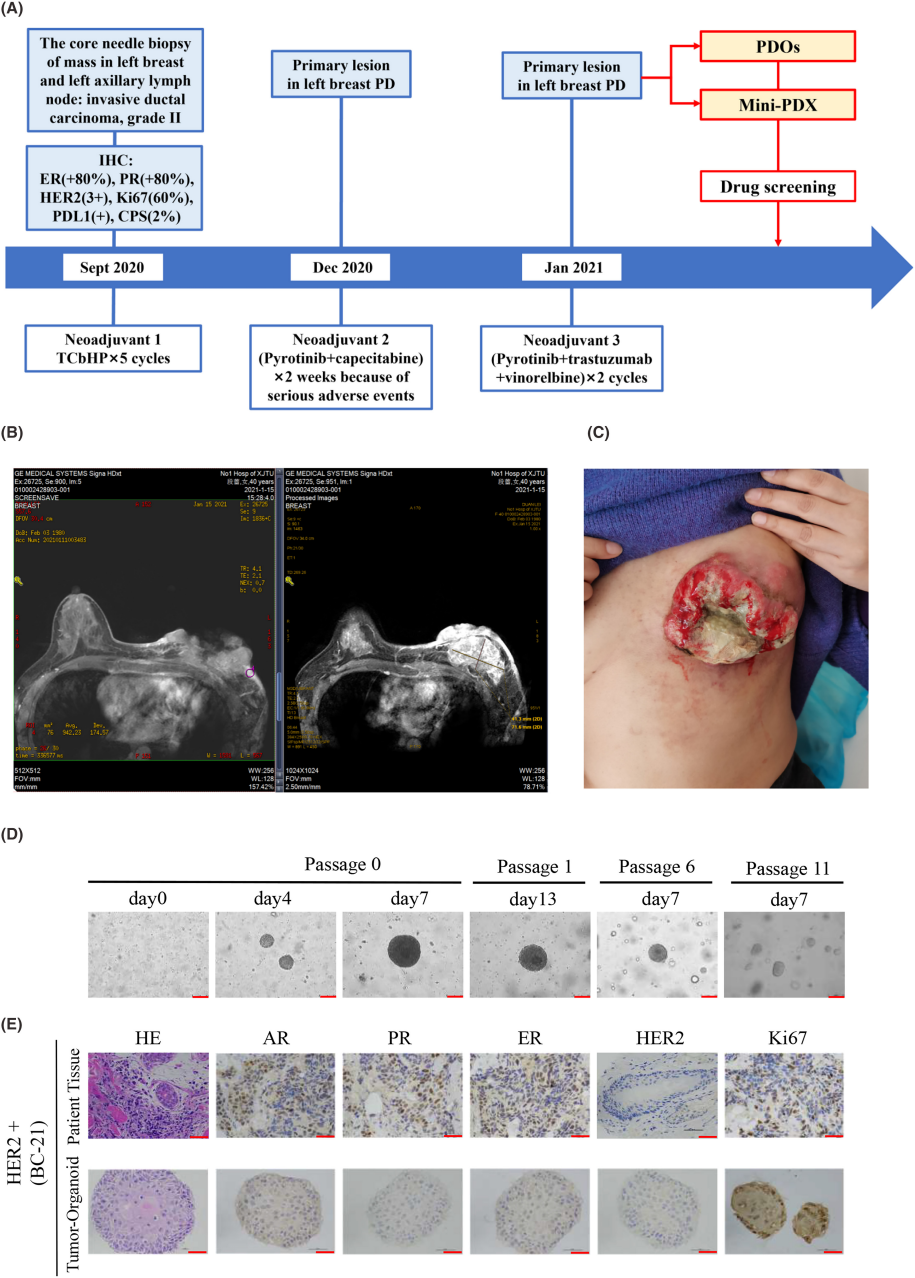
A large tumour on the left breast in a 41‐year‐old Chinese woman was observed by visual examination and MRI scan. (A) This patient's specific course of disease and treatment history. (B) Double breast MRI scan: the left breast skin has defects and multiple nodules; enhanced MRI scan of double breasts: Irregular lumps and skin nodules on the left breast showed obvious uneven enhancement. The largest cross‐section was approximately 41.3 mm × 71.6. (C) Visual examination showed that the tumour in the left breast was ulcerated and purulent. (D) The organoids were successfully established from the patient's tumour tissue by needle biopsy and can be stably passaged for more than 11 generations, Scale bar = 75 μm. (E) HE and IHC images showing the organization structure and status of breast cancer‐related markers (AR, ER, PR, HER2 and KI67) in primary tumours and organoid lines, Scale bar: 50 μm.

In August 2020, a 41‐year‐old female identified a mass in her left breast with localized erosion and blood exudate. This patient underwent a breast ultrasound at the local hospital which revealed a hypoechoic mass in the left breast, breast imaging reporting and data system (BI‐RADS) 5, and an abnormally enlarged lymph node in the left axilla, with consideration of metastasis. Pathology of the left breast and ipsilateral axillary lymph node performed based on the core needle biopsy indicated grade II invasive ductal carcinoma with the following IHC results: ER (+80%), PR (+80%), HER2 (3+) and Ki‐67 (60%). From September 2020 to December 2020, this patient received neoadjuvant chemotherapy with trastuzumab, pertuzumab, docetaxel and carboplatin (TCbHP) every 3 weeks for 4 cycles. During this treatment, the erosive wound on her left breast increased progressively. Considering disease progression, in December 2020, the treatment regimen was changed to pyrotinib and capecitabine. However, due to adverse events such as severe vomiting and diarrhoea, she stopped taking the medication on her own after 2 weeks. Later, in January 2021, the patient was admitted to the First Affiliated Hospital of Xi'an Jiaotong University for further treatment. Subsequently, the patient was administered trastuzumab, pyrotinib and vinorelbine every 3 weeks for 2 cycles (Figure 4A). However, the lesion on her left breast was still not significantly controlled. Physical examination revealed crater‐like erosion of the left breast measuring approximately 10 × 10 cm with a large amount of purulent and bloody discharge on the surface (Figure 4C). Breast magnetic resonance imaging (MRI) confirmed an irregular mass in the left breast, BIRADS 5, and left breast cancer involving the skin and left pectoralis major, with metastasis to the left axillary lymph node (cT4bN1M0, stage IIIB) (Figure 4B).

Under the condition of the patients signed informed consent, we removed a biopsy tumour sample from her left breast mass which was operated as we previously described. To explore effective antitumor drugs for this refractory patient, we successfully cultured organoids from this patient's tumour tissue. The morphology of tumour organoids showed filled lumen and dense structures when cultured for 7 and 11 days, and we stably passaged organoids to the 11th generation (Figure 4D). HE and IHC analysis showed that PDOs retained the histological characteristics of parental tissues but HER2 expression was not observed in either parental tissues or PDOs (Figure 4E).

### 3.5 In vitro drug screening for organoids in this patient

**FIGURE 5:**
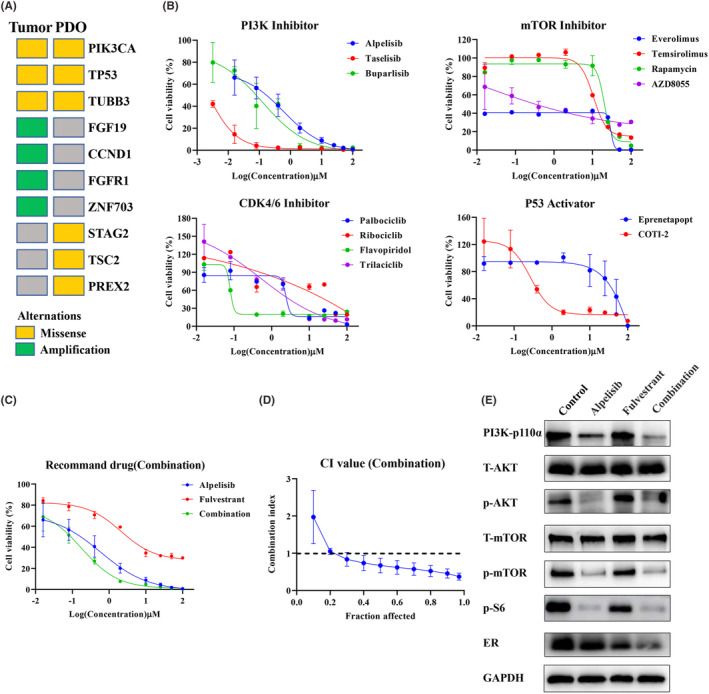
Breast cancer organoids serve as a platform for preclinical drug screening in vitro. (A) Heatmap showing that gene missense mutations in PI3K, TP53, and TUBB3 occur in both tumour tissues and organoids, amplification of FGF19, CCND1, FGFR1, and ZNF703 occurs only in tumour tissues, and missense mutations in STAG2, TSC2 and PREX2 occur only in organoids. (B) Dose–response curves including PI3K inhibitors, mTOR inhibitors, CDK4/6 inhibitors, and TP53 activators. (C) Dose–response curves of alpelisib, fulvestrant and their combination (alpelisib and fulvestrant incubated at the same time). (D) The CI values of alpelisib and fulvestrant in (C) were analysed by CompuSyn software. The mean ± SEM of results from 3 independent experiments is shown for each drug. (E) The protein expression levels of PI3K‐110α, ER, p‐AKT, T‐AKT, p‐mTOR, T‐mTOR, p‐S6 and GAPDH. p‐AKT, phosphorylated‐AKT; p‐S6, phosphorylated‐S6 protein; p‐mTOR, phosphorylated‐mTOR; T‐AKT, total‐AKT; T‐mTOR, total‐mTOR.

Based on the NGS results of the patient's PDO and parental tissue, we found that PI3KCA, TP53 and TUBB3 were mutated in both organoids and tumour tissues, indicating that the organoids were consistent with the tissue from which they were derived. In addition, PREX2, STAG2 and TSC2 were missense only in organoids and were not found in the original tumour tissue (Figure 5A). These results suggest that there is some heterogeneity in the genomic features of organoids and original tumour tissues. Later, according to the NGS results, combined with the recommended drugs of the National Comprehensive Cancer Network (NCCN) and Chinese Society of Clinical Oncology (CSCO) guidelines, a series of PDO drug screening experiments were designed and carried out in our study to find the most sensitive antitumor drugs for this patient. First, due to PI3K gene mutation, several PI3K inhibitors and mTOR inhibitors were selected for drug sensitivity testing on PDOs. The results showed that PI3K inhibitors all had good inhibitory effects (alpelisib IC50 = 0.5941 μM; taselisib IC50 = 0.0021 μM; burparlisib IC50 = 0.1261 μM), and the mTOR inhibitor AZD8055 (IC50 = 0.4646 μM) also had a good inhibitory effect (Figure 5B). In addition, TP53 activators and cyclin‐dependent kinase 4/6 (CDK4/6) inhibitors were further selected based on the TP53 mutation in the sequencing results. The results showed that the TP53 activator COTI‐2 (IC50 = 0.2725 μM) was better than APR246 (IC50 = 49.42 μM) and flavopiridol was more effective than other CDK4/6 inhibitors (Figure 5B). These results demonstrate that our established breast cancer organoids can serve as an excellent in vitro model for the screening of small molecule compounds (including drugs that are in clinical trials or have been marketed).

PI3KCA mutations are present in approximately 30%–40% of HR+/HER2‐breast cancers, which stimulate tumour growth and are associated with poor treatment response or prognosis.^24^ As the world's first oral small‐molecule α‐specific PI3K kinase inhibitor alpelisib, the NCCN guidelines recommend alpelisib in combination with fulvestrant (a selective oestrogen receptor degrader, SERD) for patients with PI3KCA‐mutated, HR+ advanced or metastatic breast cancer (grade I recommendation).^25^ Therefore, according to the actual clinical use, we further selected alpelisib, which showed a better inhibitory effect in vitro, to combine with fulvestrant. We found that the combination of the two drugs had a better effect than either durg alone, and the CI value was lower than 1, which means that the combination had a strong synergistic effect (Figure 5C,D). The PI3K pathway is a key regulator of survival, growth, proliferation, metabolism and migration.^26^ PI3K activation phosphorylates and activates AKT, which then regulates the functions of numerous cellular proteins, including the FoxO proteins, mTOR complex 1 (mTORC1) and S6 kinase.^27^ In our study, we found that alpelisib alone and the combination of alpelisib and fulvestrant can all downregulate PI3K‐p110α and its downstream signalling pathways p‐AKT, p‐mTOR and p‐S6 (Figure 5E). Consistent with previous reports, the downregulation expression of ER was observed in the fulvestrant single‐drug group, and more obviously downregulation in the combination group. These results indicated that the combination therapy of alpelisib and fulvestrant was effective against tumours through the PI3K‐AKT–mTOR signalling axis.

### 3.6 In vivo drug screening for mini‐PDX

**FIGURE 6:**
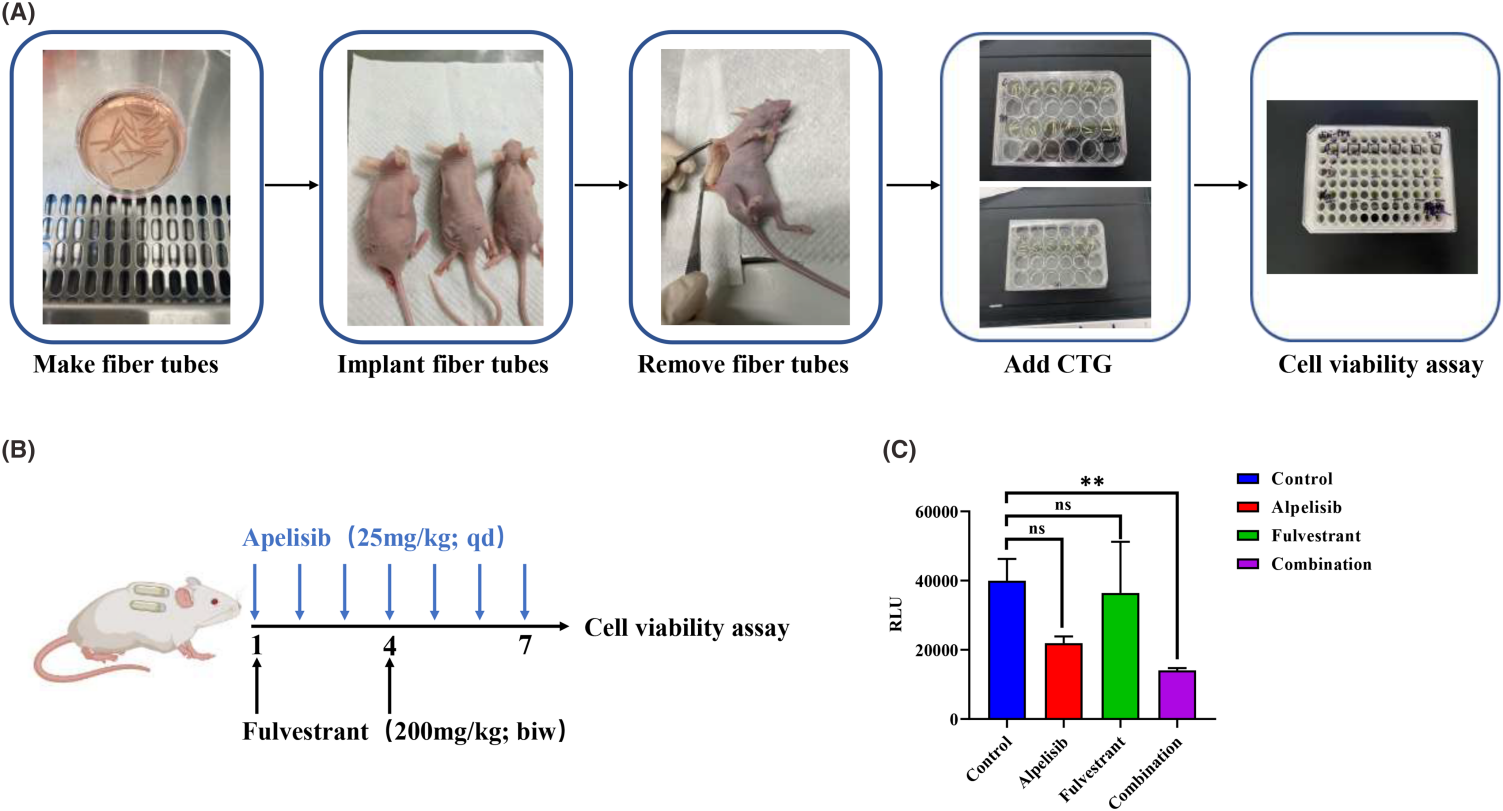
Mini‐PDX can be used as an in vivo preclinical drug screening platform to verify the in vitro screening results of PDO. (A) Detailed operation flow chart of mini‐PDX. (B) Schedule of alpelisib and fulvstrant in the mini‐PDX models: alplisib (25 mg/kg), IG (intragastric), QD (quaque die); fulvestrant (200 mg/kg), SC (subcutaneous injection), BIW (twice a week). (C) Mini‐PDX responses to alpelisib and fulvstrant in nude mice. There were three nude mice in each group, and each mouse was loaded with two hollow fibre tubes. Error bars represent the SEM of three nude mice. A *p* value <0.05 was considered significant (**p < 0.01; ns, not statistically significant).

The in vitro susceptibility test of PDOs cannot simulate the absorption, distribution, metabolism, and excretion of drugs in vivo and does not have pharmacokinetic parameters. However, the application of traditional breast cancer PDX models is limited due to the success rate, time and cost.^28^, ^29^ Therefore, we used a rapid and accurate in vivo mini‐PDX drug screening model to further verify the reliability of the in vitro drug screening results. In mini‐PDXs, the cell suspension made from the digested tumour tissue of the patient was poured into the pretreated hollow fibre tube and embedded in the subcutaneous back of the mice for 7 days. After drug administration, the viability of tumour cells in the tube was identified and in vivo drug screening results were obtained (Figure 6A). For drug treatment, alpeilisib (25 mg/kg) was gavaged orally daily for one week,^30^, ^31^, ^32^ and fulvestrant (200 mg/kg) was subcutaneously injected twice a week^33^, ^34^, ^35^ (Figure 6B). The results showed that compared with the control group, the combined group showed better synergistic inhibitory effect and the cell viability obviously decreased (Figure 6C). These results are consistent with the results of the drug screen in organoids, indicated that the combination of alpelisib and fulvestrant has a more effective antitumor effect and might provide an effective therapeutic strategy for this patient.

## 4 DISCUSSION

**FIGURE 7:**
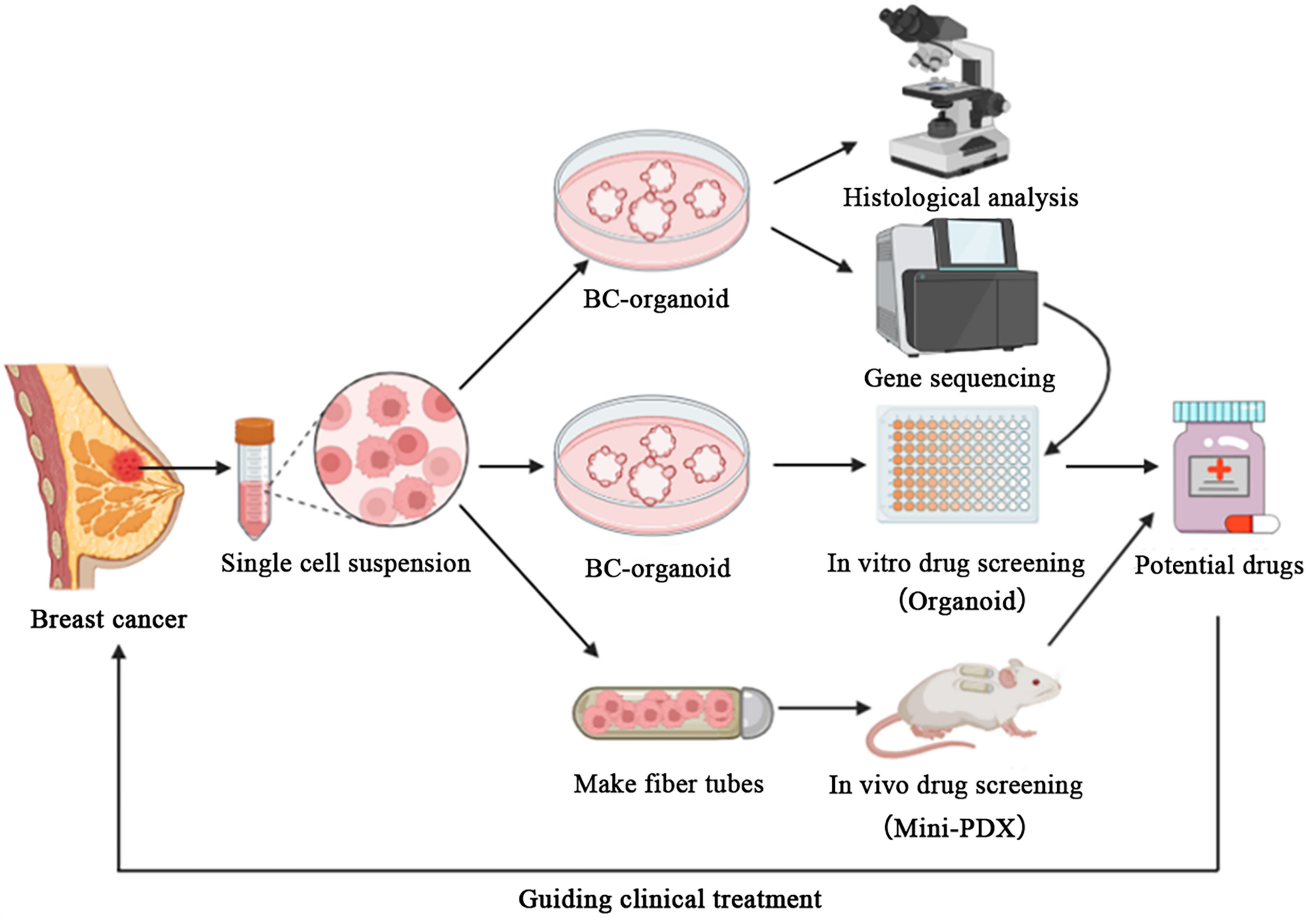
An in vitro and vivo integrated platform for accurate and rapid drug screening of breast cancer. Tumour tissue was obtained from patients and digested into cell suspensions. One part was used for in vitro studies, including PDO establishment and identification (histomorphology and genetics) and in vitro drug screening, and the other part was used for in vivo mini‐PDX studies. Combined with the results of in vivo and in vitro screening, potential therapeutic drugs were selected to guide rational clinical drug use.

Advanced breast cancer poses significant challenges due to its aggressiveness, heterogeneity and drug resistance. Developing effective treatments for these refractory cases is of utmost importance in cancer research. The PDO model has been successfully established as an in vitro cancer model in various types of cancers, including human breast cancer. However, it is inconclusive whether PDOs can guide the treatment of advanced drug‐resistant breast cancer, particularly in heavily pretreated tumours. In the present study, we established a breast cancer PDO biobank consisting of 17 breast tumour tissues and confirmed that breast cancer organoids serve as a high‐fidelity 3D in vitro model, faithfully recapitulating the histological and genetic features of the original tumours. The significance of our work lies in the first combination of PDOs with mini‐PDXs for the rapid and precise screening of drug sensitivity. To address the treatment challenges faced by a heavily pretreated advanced breast cancer patient, we employed this innovative approach to identify potential therapeutic combinations tailored to the individual. By utilizing PDOs and mini‐PDX models, we were able to develop a screening system that rapidly and accurately assesses the sensitivity of the patient's tumour to various therapeutic agents, which guides rational clinical drug use (Figure 7).

In our case, this patient developed resistance to the standard treatment regimen of multiple rounds of HER2‐targeted therapy combined with chemotherapy, resulting in rapid disease progression. HE staining of this patient's locally recurrent breast tumour and matched PDO both showed negative expression of HER2 protein. On the one hand, the intratumor heterogeneity of breast cancer is considered. On the other hand, anti‐HER2 therapy or chemotherapy can exert selective pressure on cancer cells, resulting in the emergence of drug‐resistant clones with altered HER2 status.^36^, ^37^, ^38^, ^39^

Dysregulation of HER2 downstream signalling may lead to the escape of HER2‐targeted therapy. The PI3K‐AKT–mTOR pathway is located downstream of the site where HER2‐targeted drugs act, and resistance to HER2‐targeted therapy may arise through activation of this pathway.^40^, ^41^ The PI3K‐AKT–mTOR pathway is activated via ERα and growth factor receptor (GFR) family members, which are considered to be a turntable for bidirectional crosstalk between the ER and HER2 pathways, making it a viable alternative strategy in breast cancer management.^42^ The PI3K pathway is frequently dysregulated, with activating mutations or amplifications observed in genes such as PIK3CA, which encodes the catalytic subunit of PI3K. Activating mutations of the PIK3CA gene are observed in approximately 20%–30% of HER2+ breast cancers and 30%–35% of HR+ diseases^43^ and are associated with a poorer prognosis in clinical trials of HER2‐targeted therapy compared to wild‐type PIK3CA, especially when HR is also positive. PI3K inhibitors function by inhibiting the activity of PI3K, thereby blocking downstream signalling cascades, such as AKT/mTOR, and impeding tumour growth and survival.^44^ The mechanisms of action of PI3K inhibitors are multifaceted. They induce cell cycle arrest, promote apoptosis (programmed cell death) and inhibit angiogenesis which is essential for tumour growth and metastasis. Furthermore, PI3K inhibitors have shown the potential to overcome resistance to anti‐HER2 therapies, as the PI3K pathway is known to crosstalk with the HER2 signalling pathway.^45^

We utilized matched PDOs and Mini‐PDXs for drug screening and found that the efficacy of the alpelisib was enhanced when combined with fulvestrant in this patient. Fulvestrant downregulates ER signalling, while alpelisib target the aberrant PI3K pathway. Combination therapy exerts synergistic effects by simultaneously inhibiting both pathways critical for tumour growth and survival. Unfortunately, due to the patient's financial situation and alpelisib not yet being available in China, the combination regimen cannot be applied to this patient. The combination of alpelisib and fulvestrant represents a compelling treatment strategy which targeting both the oestrogen receptor and the PI3K pathway offers the potential for enhanced efficacy and improved outcomes.

In conclusion, our study demonstrates the potential of PDO and mini‐PDX models in advancing the field of cancer research. By utilizing these models, we have made significant strides in tailoring treatment strategies for refractory breast cancer. Thus, the PDO combined with the mini‐PDX model offers a rapid and precise drug screening platform that holds promise for personalized medicine, improving patient outcomes and addressing the urgent need for effective therapies in advanced breast cancer.

The limitation of our study is the small sample size that the PDO and Mini‐PDX screening platforms were only validated in the Case report. Due to some uncontrollable factors, the combination regimen cannot be applied to this patient. However, the consistency of drug susceptibility testing in vitro and vivo which brings a new dawn for clinical patients who lack effective treatment options. Therefore, it is necessary to screen drugs for a large number of patients with advanced breast cancer, guide patients to use drugs rationally and observe the efficacy to further validate the feasibility and effectiveness of PDO and Mini‐PDX drug screening platforms.

## AUTHOR CONTRIBUTIONS

**Yuxin Cui:** Writing – original draft (equal). **Ran Ran:** Writing – original draft (equal). **Yanyan Da:** Writing – original draft (equal). **Huiwen Zhang:** Data curation (equal). **Meng Jiang:** Data curation (equal). **Xin Qi:** Data curation (equal). **Wei Zhang:** Resources (equal). **Ligang Niu:** Resources (equal). **Yuhui Zhou:** Resources (equal). **Can Zhou:** Resources (equal). **Xiaojiang Tang:** Resources (equal). **Ke Wang:** Resources (equal). **Yu Yan:** Resources (equal). **Yu Ren:** Resources (equal). **Danfeng Dong:** Investigation (equal). **Yan Zhou:** Methodology (equal). **Hui Wang:** Investigation (equal). **Jin Gong:** Data curation (equal). **Fang Hu:** Formal analysis (equal). **Shidi Zhao:** Formal analysis (equal). **Huimin Zhang:** Resources (equal); supervision (equal). **Chengsheng Zhang:** Supervision (equal). **Jin Yang:** Supervision (equal); writing – review and editing (equal).

## FUNDING INFORMATION

This study was supported by the Key Clinical Research Project of the First Affiliated Hospital of Xi'an Jiaotong University (XJTU1AF‐CRF‐2020‐006) and the National Health Commission of People's Republic of China on the projects entitled ‘Multidisciplinary cooperation on diagnosis and treatment of gastric cancer’ (2019–2024) (QT252) and ‘Development of scientific system and service platform for cancer precision medicine’ (2020–2025) (TQ264) and Youth Project of Shaanxi Natural Science Foundation (2024JC‐YBQN‐0932).

## CONFLICT OF INTEREST STATEMENT

The authors have no conflicts of interest to declare.

## Supporting information


Table S1.

## Data Availability

The datasets used and/or analysed during the current study are available from the corresponding author on reasonable request.

## References

[1] Sung H , Ferlay J , Siegel RL , et al. Global cancer statistics 2020: GLOBOCAN estimates of incidence and mortality worldwide for 36 cancers in 185 countries. CA Cancer J Clin. 2021;71(3):209‐249. 10.3322/caac.21660 33538338

[2] Gonzalez‐Angulo AM , Morales‐Vasquez F , Hortobagyi GN . Overview of resistance to systemic therapy in patients with breast cancer. Adv Exp Med Biol. 2007;608:1‐22. 17993229 10.1007/978-0-387-74039-3_1

[3] Hayat MJ , Howlader N , Reichman ME , Edwards BK . Cancer statistics, trends, and multiple primary cancer analyses from the surveillance, epidemiology, and end results (SEER) program. Oncologist. 2007;12(1):20‐37. 10.1634/theoncologist.12-1-20 17227898

[4] Yang LP , Liu BE , Chen HD , et al. Progress in the application of organoids to breast cancer research. J Cell Mol Med. 2020;24(10):5420‐5427. 10.1111/jcmm.15216 32283573 PMC7214171

[5] Testa U , Castelli G , Pelosi E . Breast cancer: a molecularly heterogenous disease needing subtype‐specific treatments. Med Sci (Basel). 2020;8(1):18. doi:10.3390/medsci8010018 32210163 PMC7151639

[6] Murayama T , Gotoh N . Patient‐derived xenograft models of breast cancer and their application. Cells‐Basel. 2019;8:8. doi:10.3390/cells8060621 PMC6628218 31226846

[7] Campaner E , Zannini A , Santorsola M , et al. Breast cancer organoids model patient‐specific response to drug treatment. Cancer. 2020;12(12):3869. doi:10.3390/cancers12123869 PMC7770601 33371412

[8] Ge Y , Zhang X , Liang W , et al. OncoVee™‐MiniPDX‐guided anticancer treatment for gastric cancer patients with synchronous liver metastases: a retrospective cohort analysis. Front Oncol. 2021;11:757383. doi:10.3389/fonc.2021.757383 35047388 PMC8761725

[9] Zhu XD , Xu XQ , Zhang BA , et al. Individualized therapy based on the combination of mini‐PDX and NGS for a patient with metastatic AFP‐producing and HER‐2 amplified gastric cancer. Oncol Lett. 2022;24(5):411. doi:10.3892/ol.2022.13531 36245818 PMC9554957

[10] Xu J , Gao Y , Luan XT , et al. An effective AKT inhibitor‐PARP inhibitor combination therapy for recurrent ovarian cancer. Cancer Chemother Pharmacol. 2022;89(5):683‐695. doi:10.1007/s00280-022-04403-9 35419627 PMC9054880

[11] Huang YK , Xu J , Li K , Wang J , Dai YL , Kang Y . A novel, personalized drug‐screening system for platinum‐resistant ovarian cancer patients: a preliminary clinical report. Cancer Manag Res. 2021;13:2849‐2867. doi:10.2147/Cmar.S276799 33833569 PMC8020460

[12] Zhan M , Yang RM , Wang H , et al. Guided chemotherapy based on patient‐derived mini‐xenograft models improves survival of gallbladder carcinoma patients. Cancer Commun. 2018;38:38. doi:10.1186/s40880-018-0318-8 PMC6050666 30016995

[13] Liu SL , Liang HB , Yang ZY , et al. Gemcitabine and XCT790, an ERRα inverse agonist, display a synergistic anticancer effect in pancreatic cancer. Int J Med Sci. 2022;19(2):286‐298. doi:10.7150/ijms.68404 35165514 PMC8795805

[14] Sachs N , de Ligt J , Kopper O , et al. A living biobank of breast cancer organoids captures disease heterogeneity. Cell. 2018;172:373‐386.e10. doi:10.1016/j.cell.2017.11.010 29224780

[15] Yang Z , Yang N , Ou QX , et al. Investigating novel resistance mechanisms to third‐generation EGFR tyrosine kinase inhibitor Osimertinib in non‐small cell lung cancer patients. Clin Cancer Res. 2018;24(13):3097‐3107. doi:10.1158/1078-0432.Ccr-17-2310 29506987

[16] Chen P , Zhang X , Ding RB , et al. Patient‐derived organoids can guide personalized‐therapies for patients with advanced breast cancer. Adv Sci. 2021;8(22):e2101176. doi:10.1002/advs.202101176 PMC8596108 34605222

[17] Pan B , Zhao DY , Liu YQ , et al. Establishment and characterization of breast cancer organoids from a patient with mammary Paget's disease. Cancer Cell Int. 2020;20(1):365. doi:10.1186/s12935-020-01459-6 32774159 PMC7397673

[18] Goldhammer N , Kim J , Timmermans‐Wielenga V , Petersen OW . Characterization of organoid cultured human breast cancer. Breast Cancer Res. 2019;21(1):141. doi:10.1186/s13058-019-1233-x 31829259 PMC6907265

[19] Lebeau A , Denkert C . Updated WHO classification of tumors of the breast: the most important changes. Pathologe. 2021;42(3):270‐280. doi:10.1007/s00292-021-00934-9 33822251

[20] Zhang XP , Yang HJ , Zhang RP . Challenges and future of precision medicine strategies for breast cancer based on a database on drug reactions. Biosci Rep. 2019;39(9). doi:10.1042/Bsr20190230 PMC6732363 31387972

[21] Aggelis V , Johnston SRD . Advances in endocrine‐based therapies for estrogen receptor‐positive metastatic breast cancer. Drugs. 2019;79(17):1849‐1866. doi:10.1007/s40265-019-01208-8 31630379

[22] Wang RX , Chen S , Jin X , Chen CM , Shao ZM . Weekly paclitaxel plus carboplatin with or without trastuzumab as neoadjuvant chemotherapy for HER2‐positive breast cancer: loss of HER2 amplification and its impact on response and prognosis. Breast Cancer Res Tr. 2017;161(2):259‐267. doi:10.1007/s10549-016-4064-9 27885439

[23] Ishiguro T , Ohata H , Sato A , Yamawaki K , Enomoto T , Okamoto K . Tumor‐derived spheroids: relevance to cancer stem cells and clinical applications. Cancer Sci. 2017;108(3):283‐289. 10.1111/cas.13155 28064442 PMC5378268

[24] Miricescu D , Totan A , Stanescu‐Spinu II , Badoiu SC , Stefani C , Greabu M . PI3K/AKT/mTOR signaling pathway in breast cancer: from molecular landscape to clinical aspects. Int J Mol Sci. 2020;22(1):173. doi:10.3390/ijms22010173 33375317 PMC7796017

[25] Vernieri C , Corti F , Nichetti F , et al. Everolimus versus alpelisib in advanced hormone receptor‐positive HER2‐negative breast cancer: targeting different nodes of the PI3K/AKT/mTORC1 pathway with different clinical implications. Breast Cancer Res. 2020;22(1):33. doi:10.1186/s13058-020-01271-0 32252811 PMC7137211

[26] Alzahrani AS . PI3K/Akt/mTOR inhibitors in cancer: At the bench and bedside. Semin Cancer Biol. 2019;59:125‐132. doi:10.1016/j.semcancer.2019.07.009 31323288

[27] Dibble CC , Cantley LC . Regulation of mTORC1 by PI3K signaling. Trends Cell Biol. 2015;25(9):545‐555. doi:10.1016/j.tcb.2015.06.002 26159692 PMC4734635

[28] Dobrolecki LE , Airhart SD , Alferez DG , et al. Patient‐derived xenograft (PDX) models in basic and translational breast cancer research. Cancer Metastasis Rev. 2016;35(4):547‐573. doi:10.1007/s10555-016-9653-x. 28025748 PMC5396460

[29] Na D , Moon HG . Patient‐derived xenograft models in breast cancer research. Trans Res Breast Canc. 2021;1187:283‐301. doi:10.1007/978-981-32-9620-6_14 33983584

[30] Fritsch C , Huang A , Chatenay‐Rivauday C , et al. Characterization of the novel and specific PI3Kα inhibitor NVP‐BYL719 and development of the patient stratification strategy for clinical trials. Mol Cancer Ther. 2014;13(5):1117‐1129. doi:10.1158/1535-7163.Mct-13-0865 24608574

[31] Gobin B , Baud'Huin M , Lamoureux F , et al. BYL719, a new α‐specific PI3K inhibitor: Single administration and in combination with conventional chemotherapy for the treatment of osteosarcoma. Int J Cancer. 2015;136(4):784‐796. doi:10.1002/ijc.29040 24961790

[32] Novoplansky O , Shnerb AB , Marripati D , et al. Activation of the EGFR/PI3K/AKT pathway limits the efficacy of trametinib treatment in head and neck cancer. Mol Oncol. 2023;17:2618‐2636. doi:10.1002/1878-0261.13500 37501404 PMC10701778

[33] Servetto A , Kollipara R , Formisano L , et al. Nuclear FGFR1 Regulates Gene Transcription and Promotes Antiestrogen Resistance in ER Breast Cancer. Clin Cancer Res. 2021;27(15):4379‐4396. doi:10.1158/1078-0432.Ccr-20-3905 34011560 PMC8338892

[34] Bernoulli J , Konkol Y , Vuorikoski H , Yatkin E . Effects of Afala and antiestrogen ICI 182,780 in the model of hormone‐dependent prostate inflammation. B Exp biol med. 2014;156(6):807‐809. doi:10.1007/s10517-014-2456-6 24824703

[35] Gancarczyk M , Paziewska‐Hejmej A , Carreau S , Tabarowski M , Bilinska B . Dose‐ and photoperiod‐dependent effects of 17β‐estradiol and the anti‐estrogen ICI 182,780 on testicular structure, acceleration of spermatogenesis, and aromatase immunoexpression in immature bank voles. Acta Histochem. 2004;106(4):269‐278. doi:10.1016/j.acthis.2004.04.002 15350809

[36] Katayama A , Miligy IM , Shiino S , et al. Predictors of pathological complete response to neoadjuvant treatment and changes to post‐neoadjuvant HER2 status in HER2‐positive invasive breast cancer. Mod Pathol. 2021;34(7):1271‐1281. doi:10.1038/s41379-021-00738-5 33526875 PMC8216906

[37] Li PF , Liu TT , Wang YM , et al. Influence of Neoadjuvant chemotherapy on HER2/ status in invasive breast cancer. Clin Breast Cancer. 2013;13(1):53‐60. doi:10.1016/j.clbc.2012.09.011 23103368

[38] Niikura N , Tomotaki A , Miyata H , et al. Changes in tumor expression of HER2 and hormone receptors status after neoadjuvant chemotherapy in 21,755 patients from the Japanese breast cancer registry. Ann Oncol. 2016;27(3):480‐487. doi:10.1093/annonc/mdv611 26704052

[39] Gahlaut R , Bennett A , Fatayer H , et al. Effect of neoadjuvant chemotherapy on breast cancer phenotype, ER/PR and HER2 expression—implications for the practising oncologist. Eur J Cancer. 2016;60:40‐48. doi:10.1016/j.ejca.2016.03.006 27062316

[40] Nunnery SE , Mayer IA . Targeting the PI3K/AKT/mTOR pathway in hormone‐positive breast cancer. Drugs. 2020;80(16):1685‐1697. doi:10.1007/s40265-020-01394-w 32894420 PMC7572750

[41] Jacquemetton J , Kassem L , Poulard C , et al. Analysis of genomic and non‐genomic signaling of estrogen receptor in PDX models of breast cancer treated with a combination of the PI3K inhibitor alpelisib (BYL719) and fulvestrant. Breast Cancer Res. 2021;23(1):57. doi:10.1186/s13058-021-01433-8 34020697 PMC8139055

[42] Sanchez CG , Ma CX , Crowder RJ , et al. Preclinical modeling of combined phosphatidylinositol‐3‐kinase inhibition with endocrine therapy for estrogen receptor‐positive breast cancer. Breast Cancer Res. 2011;13(2):R21. doi:10.1186/bcr2833 21362200 PMC3219179

[43] Reinhardt K , Stückrath K , Hartung C , et al. Mutations in breast cancer. Breast Cancer Res Tr. 2022;196(3):483‐493. doi:10.1007/s10549-022-06637-w PMC9633529 36279023

[44] Ellis H , Ma CX . PI3K Inhibitors in Breast Cancer Therapy. Curr Oncol Rep. 2019;21(12):110. doi:10.1007/s11912-019-0846-7 31828441

[45] Crowder RJ , Ellis MJ . Treating breast cancer through novel inhibitors of the phosphatidylinositol 3′‐kinase pathway. Breast Cancer Res. 2005;7(5):212‐214. doi:10.1186/bcr1307 16168140 PMC1242159

