# Supplementary figures and images for: The combination of breast cancer PDO and mini‐PDX platform for drug screening and individualized treatment
Source: J Cell Mol Med. 2024 May 9;28(9):e18374. doi: 10.1111/jcmm.18374 (PMC11081008; doi:10.1111/jcmm.18374)

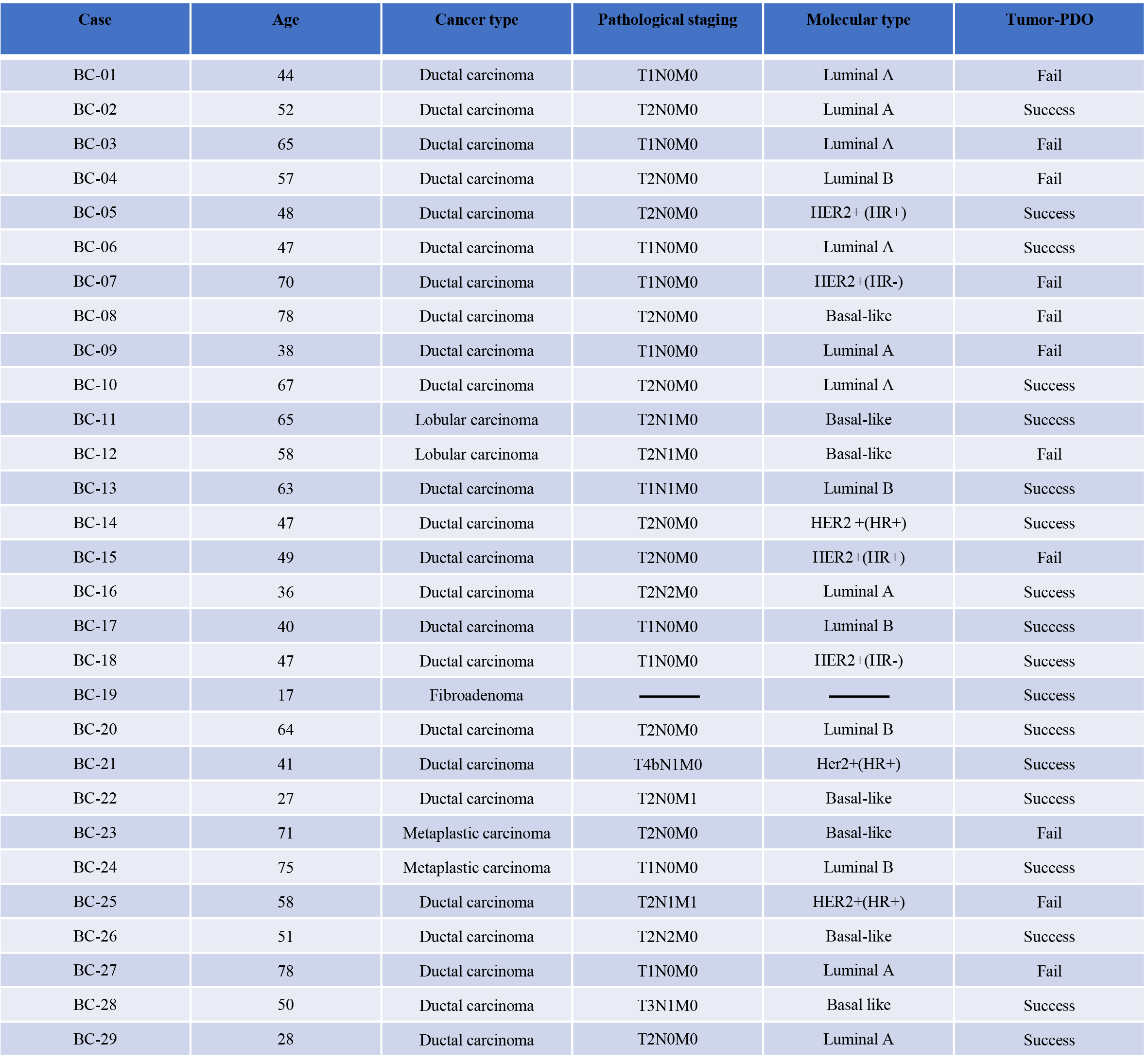

Supplement: Supplementary file 1 — Table S1. [file JCMM-28-e18374-s001.tif]
